# Hyaluronidase-induced matrix remodeling contributes to long-term synaptic changes

**DOI:** 10.3389/fncir.2024.1441280

**Published:** 2025-01-17

**Authors:** Rostislav Sokolov, Viktoriya Krut', Vsevolod Belousov, Andrey Rozov, Irina V. Mukhina

**Affiliations:** ^1^Institute of Biology and Biomedicine, Lobachevsky State University of Nizhny Novgorod, Nizhny Novgorod, Russia; ^2^Pirogov Russian National Research Medical University, Moscow, Russia; ^3^Federal Center of Brain Research and Neurotechnologies, Federal Medical Biological Agency, Moscow, Russia; ^4^Shemyakin-Ovchinnikov Institute of Bioorganic Chemistry, Russian Academy of Sciences, Moscow, Russia; ^5^Life Improvement by Future Technologies (LIFT) Center, Moscow, Russia; ^6^Institute of Fundamental Medicine, Privolzhsky Research Medical University, Nizhny Novgorod, Russia

**Keywords:** hippocampus, extracellular matrix, synaptic plasticity, NMDA receptors, hyaluronidase

## Abstract

Extracellular brain space contains water, dissolved ions, and multiple other signaling molecules. The neural extracellular matrix (ECM) is also a significant component of the extracellular space. The ECM is synthesized by neurons, astrocytes, and other types of cells. Hyaluronan, a hyaluronic acid polymer, is a key component of the ECM. The functions of hyaluronan include barrier functions and signaling. In this article, we investigate physiological processes during the acute phase of enzymatic ECM removal. We found that hyaluronidase, an ECM removal agent, triggers simultaneous membrane depolarization and sharp calcium influx into neurons. Spontaneous action potential firing frequency increased rapidly after ECM destruction in interneurons, but not pyramidal neurons. Hyaluronidase-dependent calcium entry can be blocked by a selective antagonist of N-methyl-D-aspartate (NMDA) receptors, revealing these receptors as the main player in the observed phenomenon. Additionally, we demonstrate increased NMDA-dependent long-term potentiation at CA3-to-CA1 synapses during the acute phase of ECM removal. These findings suggest that hyaluronan is a significant synaptic player.

## Introduction

In the 1890s, Ramon y Cajal and Golgi first described brain extracellular space. Living tissue is considered as a dynamic meshwork of cells and fluids. The extracellular matrix (ECM) acts as a filler between cells in the tissue. It is also filled with water and various dissolved substances that occupy the extracellular space. It is now known that the ECM is an organized network of macromolecules and occupies approximately 20% of the volume of the adult brain (Wolak and Thorne, 2013). For a long time, it was considered that the only function of the ECM is to maintain the structure of the brain due to the presence of cell adhesion molecules (Storms and Rutishauser, 1998). The structure of the ECM is heterogeneous and consists of regulatory proteins and polysaccharides, synthesized by various types of cells: glycosaminoglycans (hyaluronic acid), proteoglycans (neurocan, brevican, etc.), glycoproteins (tenascins), and collagen (Dankovich et al., 2021; Dityatev et al., 2021; Soles et al., 2023). Recently, researchers have discovered new functions of the ECM. For instance, the barrier functions of the ECM (diffusion, ion solubility, water retention, and cell migration) have been outlined (Joerges et al., 2012; Georgi et al., 2019; Amin and Borrell, 2020). At the same time, it is essential to consider the ECM as a modulator of fundamental signaling processes during brain ontogenesis and in adulthood (neurite growth, synaptogenesis, plasticity) (Horn et al., 2007; Kochlamazashvili et al., 2010; Lam et al., 2019; Wilson et al., 2020; Long and Huttner, 2021). For a long time, the ECM was regarded as a stable structure with limited variability. However, there are both mobile and immobile ECM structures with varying turnover of components. It has recently been shown that some matrix components, such as tenascin-R, undergo fast endocytosis and resurfacing over 18 h (Dankovich et al., 2021). The hyaluronic acid receptor for endocytosis (HARE; also designated Stabilin-2) mediates the systemic clearance of hyaluronan and chondroitin sulfates. This receptor has a recycling time of approximately 12 min. Unfractioned heparin (3000–30,000 Da) has a half-life of approximately 1 h, while low-molecular-weight heparin (300–8,000 Da) has a half-life ranging from 3 h to 17 h(Harris et al., 2008). An estimated one-third of total body hyaluronic acid (HA) (∼15 g in a person with a body weight of 70 kg) is turned over daily (Tobisawa et al., 2021; Fraser and Laurent, 1989).

It has been shown that hyaluronic acid polymer (hyaluronan) is a key component of the ECM (Day and Sheehan, 2001). The function of hyaluronan is to anchor ECM components to each other and to tenascin proteins. Furthermore, hyaluronan and hyaluronic acid can directly interact with the membrane proteins (*β*-integrin, receptor for hyaluronan mediated motility (RHAMM) or CD168, CD44, toll-like receptors) (Nagy et al., 1995; Jiang et al., 2011; Ferrari et al., 2016; Schweitzer et al., 2017). There is not much information on endogenous brain hyaluronidase (Margolis et al., 1972; Chowdhury et al., 2016). The removal of brain ECM by an exogenous enzyme was primarily considered as a model of acute rapid change of the state of the ECM. The literature suggests the ECM may undergo remodeling in animals due to stress conditions, aging, and the presence of neurodegenerative diseases. Additionally, microglial cells are regulators of ECM clearance and spine formation (Nguyen et al., 2020). There are products of ECM degradation called matrikines and matricryptins, which are bioactive fragments released from the ECM by enzymatic degradation (Ricard-Blum and Salza, 2014). They regulate numerous physiological and pathological processes (Collins et al., 2011). Hyaluronan is also capable of generating matricryptins during its degradation, thereby releasing ECM components that are anchored to hyaluronan (Clerc et al., 2019).

The ECM that surrounds neurons, also referred to as perineuronal nets (PNNs), plays an important role in synaptic stabilization and plasticity during development (Pizzorusso et al., 2002; Nowicka et al., 2009). Hyaluronan is identified in the PNNs surrounding neuronal bodies, dendrites, and synapses. The primary component of PNNs is hyaluronic acid. One approach to address the function of the ECM is through enzymatic ECM removal with specific enzymes such as hyaluronidase (Hyase), chondroitinase ABC, and heparinase. The *in vitro* removal of highly sulfated heparan sulfates with heparinase reduced CA1 pyramidal cell excitability, decreased Ca^2+^ entry into dendritic spines, and impaired long-term potentiation (LTP) (Minge et al., 2017). Acute treatment with chondroitinase ABC did not affect the excitability of fast-spiking interneurons (FSIs) and CA2 pyramidal neurons; however, 7 days after *in vivo* injection of the enzyme, an increase in FSI and PC excitability was observed along with a reduction in excitatory transmission and an increase in inhibitory transmission to FSIs (Hayani et al., 2018). Other researchers found that mice injected in the hippocampus with hyaluronidase exhibited an increase in audiogenic seizures, along with an enrichment of proinflammatory pathways (Balashova et al., 2019). Hyaluronan is present at excitatory synapses (Wilson et al., 2020). Additionally, hyaluronan is known to change the activity of L-type voltage-dependent calcium channels (L-VDCC), increase endogenous GluR1-containing *α*-amino-3-hydroxy-5-methyl-4-isoxazolepropionic acid AMPA receptor surface mobility, and increase the surface expression of NR2b, a subunit of N-methyl-D-aspartate NMDA receptors (Kochlamazashvili et al., 2010; Schweitzer et al., 2017; Frischknecht et al., 2009). It is logical that as a result of the destruction of the ECM, a decrease in LTP occurs (Kochlamazashvili et al., 2010; Minge et al., 2017; Hayani et al., 2018). A hyaluronidase-dependent model of epilepsy was previously characterized in cell cultures (Vedunova et al., 2013). Hyaluronan synthase 3 (HAS3) is known to be the protein responsible for the production of hyaluronan in the brain (Csoka et al., 2001). HAS3 knockout mice have an epileptic phenotype (Arranz et al., 2014). However, most studies focus on the delayed (from several hours to several days) effects of ECM removal on cellular and network activity, and the immediate effects occurring during the acute phase of ECM attenuation are still unknown.

Here, we demonstrate the effects of acute remodeling of the ECM with hyaluronidase. First, we used primary cell cultures of the mouse hippocampus. In response to the application of hyaluronidase, we observed rapid calcium entry as well as neuronal membrane potential depolarization. We screened the expected molecular targets with selective blockers, attempting to attenuate the calcium entry. With this approach, we identified the target responsible for the prolonged calcium entry accompanied by membrane depolarization. The results obtained in the cell culture model were replicated in acute mouse hippocampal slices. Following this, we evaluated the effect of rapid ECM removal on hippocampal synaptic plasticity. We demonstrated that short-lasting disintegration of the ECM increased NMDA-dependent LTP at CA3-to-CA1 synapses.

## Materials and methods

Comprehensive information about the materials and methods used in this study is provided in the supplementary file.

### Animal statement

All experiments were conducted in accordance with the European Convention 1986 86/609/EEC. The protocol for mice cell culture preparations and mice acute brain slice preparation was approved by the Ethical Committee of Pirogov Russian National Research Medical University. C57BL/6 J strain mice were used in this study. The mice had ad libitum access to food and sterilized water.

### Cell culture preparation

Mixed primary hippocampal cell cultures were prepared from E18 embryos or P0 pups without differentiating by sex. For astrocyte cultures, Dulbecco’s modified Eagle medium (DMEM) was used instead of neurobasal media. Another key difference in astrocyte cultivation was the increased percentage of fetal bovine serum (FBS) (10% during the first week and 2.5% during the remainder of the cultivation period). For cell culture preparation and cultivation, Thermo Scientific mediums and supplements (Gibco, USA) were used. Cultures were prepared in accordance with details provided in (Sokolov and Mukhina, 2022). Experimental procedures started from the 21st day *in vitro* (DIV). The names of the chemicals used with the catalog number are provided in Supplementary Table 1.

### Construction of vectors for expression of GCaMP6f,s

To express GCaMP6f in neurons in vitro and GCaMP6s in astrocytes in vitro, we created recombinant adeno-associated viral (AAV) vectors, carrying the respective genetic constructs. The plasmid containing hSyn promoter—the promoter of the human gene synapsin 1—was provided by the Penn Vector Core (Addgene plasmid #100848). The plasmid containing GCaMP6f was provided by Balijit Khakh (Addgene plasmid #52925).

The construct pAAV-hSyn-GCamp6f-SV40 was created on the basis of pAAV.Syn.NES.jCaMP1a.WPRE.SV40 and pZac2.1gfaABC1D-cyto-GCaMP6f by replacing a promoter and fusing the corresponding cDNAs in the same open read frame. All manipulations were conducted between the NheI and MfeI restriction enzyme sites. For the expression of GCaMP6s in astrocytes, we used a recombinant AAV-9 carrying the construct pAAV-GFAP-GCamp6s-(wpre-sv40), where GFAP is a promoter of glial fibrillar acid protein. The constructs were packaged into AAV-9 viral particles for astrocytes and AAV-DJ for neurons at the Viral Core Facility of the Shemyakin-Ovchinnikov Institute of Bioorganic Chemistry.

### Extracellular brain matrix destruction by hyaluronidase treatment of cultures and slices

For ECM destruction, we used hyaluronidase from bovine testes (Hyase, Sigma-Aldrich, H3506-1G, 400-1000 U/mg). The final concentration of 0.1 mg/mL (0.04–0.1 U/μl) was used for culture treatment. The final concentration of 0.14 mg/mL (0.06–0.14 U/μl) was used for slice treatment.

### Intracellular calcium recordings and analysis

For intracellular calcium recordings, we used Oregon Green-488 BAPTA-1 AM (OGB1, Invitrogen, USA) or GCaMP6f calcium sensors for neurons. For intracellular calcium recordings of astrocytes, we used GCaMP6s. The calcium sensor used is specified in the figures and in the text. For detailed OGB1 measurements, see Sokolov and Mukhina (2022). Neurons were viewed and acquired with the same setup with an ORCA Flash4.0 LT plus digital sCMOS camera (RRID: SCR_021971, Hamamatsu Photonics, Hamamatsu City, Japan). Data acquisition and region of interest (ROI) positioning were performed using the free software uManager (RRID: SCR_000415) at 20 fps. Offline measurements of time-dependent changes in fluorescence intensity were performed using Fiji software (Schindelin et al., 2012) (RRID: SCR_002285). The analysis of fluorescent calcium sensors was performed only in neurons with neuronal spontaneous calcium activity.

### Electrophysiological recordings of cultured neurons

Electrophysiological recordings of cultured neurons were conducted at 21–26 DIV. Patch electrodes were pulled from hard borosilicate capillary glass [Sutter P-97/PC Pipette Puller (RRID: SCR_018636)]. Whole-cell patch-clamp recordings were obtained at room temperature (23–25°C) using pipettes (2–3 MΩ) that were filled with 130 mM K-gluconate, 1 mM MgCl_2_, 3 mM L-ascorbic acid, 10 mM HEPES, 2.5 mM Na_2_ATP, 1 mM Na_3_GTP, and adjusted to 295 ± 3 mOsm, with a pH of 7.35. Recordings were obtained at room temperature (23–25°C) and the perfusion solution contained 130 mM NaCl, 2.5 mM KCl, 1.5 mM MgCl_2_, 1.5 mM CaCl_2_ 10 mM glucose, 10 mM HEPES, pH 7.33 (HEPES-based solution). A Heka Elektronik EPC 10 USB Patch-Clamp Amplifier (RRID: SCR_018399) was connected to computer-running Patchmaster software (RRID: SCR_000034).

### Acute brain slice electrophysiological procedures

C57BL/6 J strain male mice P30-45 were used for acute brain slice preparation. The preparation of acute brain slices was performed as in Kalinichenko et al. (2023). During electrophysiological recordings, acute brain slices were continuously perfused with artificial cerebrospinal fluid (ACSF) containing in 125 mM NaCl, 25 mM NaHCO_3_, 2.5 mM KCl, 1.25 mM NaH2PO_4_, 1 mM MgCl_2_, 2 mM CaCl_2_, and 25 mM D-glucose bubbled with 95% O_2_ and 5% CO_2_.

Electrodes for the postsynaptic pyramidal cells were filled with a solution consisting of 136 mM Cs-gluconate, 4 mM CsCl, 10 mM HEPES, 8 mM NaCl, 4 mM MgATP, 03 mM MgGTP, and 10 mM phosphocreatine (pH 7.3 with CsOH). Whole-cell recordings from neurons were conducted at room temperature (23–25°C) in a voltage-clamp mode. CA1 pyramidal cells were visually identified using DIC at 40× magnification. GABAergic synaptic transmission was blocked by the continuous presence of the GABAA receptor antagonist SR95531 (10 μM). LTP procedures were performed in accordance with (Kalinichenko et al., 2023; Rozov et al., 2012). Briefly, the statistical significance of LTP for slices that received Hyase treatment under different experimental conditions was assessed by comparing normalized excitatory postsynaptic currents (EPSC) amplitudes in the paired apical and unpaired basal pathways recorded in the period 0–30 min after LTP induction. For cross-comparison effects of treated and untreated neurons, the values of relative potentiation calculated by subtraction of the normalized EPSC amplitudes in the unpaired basal pathway from those in the paired apical pathway were used. For NMDARs, evoked EPSCs were measured in the apical pathway. We used ACSF without magnesium, supplemented with 50 μM magnesium chelator EDTA. The baseline was measured in the presence of the GABAA receptor antagonist SR95531 (10 μM) and AMPAR blocker CNQX. After Hyase treatment and recording, NMDAR EPSCs were blocked with D-2-Amino-5-phosphonovalerate (D-APV).

### Extracellular matrix *in vitro* recordings

WFA-Alexa594 was added to the culture media and the cultures were incubated at +37C (5% CO_2_) for 30 min. The final concentration of WFA was 6.5 μg/mL. The solution was removed, and the cultures were washed with 1 mL of warm HEPES-based solution, after which the dish was filled with 3 mL of HEPES-based solution. Live-cell fluorescent imaging of primary neuronal cell cultures labeled with WFA-Alexa594 was carried out using a Nikon ECLIPSE Ti2-E epifluorescent microscope equipped with a SPECTRA X light engine and a Photometrics BSI camera. The fluorescent signals of AlexaFluor594 were acquired using Plan Apo *λ* 40x (NA 0.95) objectives. Live cell imaging was carried out using Nikon NIS-Elements software. AlexaFluor594 was excited at a 555 nm, and its fluorescence was acquired within the spectral range 580–610 nm. We performed time-lapse imaging with 10-s interframe interval and 3-s exposure to measure changes in fluorescence in neurons (red positive cells). During the 10-s interframe interval, HEPES-based solution, inactive hyaluronidase, and active hyaluronidase were added. Images were captured in two randomly selected fields of view for each dish.

### Slice preparations for WFA staining

For brain tissue dissection, fixation, and slicing, prior to WFA slice staining, mice were transcardially perfused with 4% PFA. Then, the brains were dehydrated using a sucrose gradient. First, 50 μm slices were cut on a HM525 NX cryotome (Thermo Scientific, USA) and stored in PBS-azide solution. For staining, slices were incubated in 25 mM glycine solution for 20 min and then washed three times with PBS. Then, Hyase was added to the slices for 1 h. The slices were washed three times with PBS. The slices were initially permeabilized in PBS with 0.5% Triton X-100 (Sigma-Aldrich, #T8787) for 1 h. Blocking was carried out in PBS containing 5% goat serum (Sigma- Aldrich, #G9023) and 0.1% Triton X-100 for 1 h. Then, the slices were stained with WFA-FITC (20 ug/ml), diluted in the blocking solution (0.1% triton, 1% goat serum) for 48 h at 4°C. After staining, the slices were washed three times with PBS (5 min each), mounted on slides, and visualized under a Nikon A1 confocal microscope.

### Primary culture immunostaining with antibodies

Immunostaining was performed starting at 24 DIV. Cultures were washed with PBS (35°С). Next, the cultures were fixed for 15 min with fresh 4% PFA at room temperature. After fixation, the cultures were washed three times with PBS for 5 min each. Permeabilization was performed in PBS with 1% Triton X-100 for 15 min. Blocking was carried out in PBS containing 5% goat serum (Sigma-Aldrich, #G9023) and 0.1% Triton X-100 for 1 h. Then cultures were washed with PBS and 0.1% Triton X-100. Primary antibody labeling was carried for the next 12 h at 4°С in a blocking solution, containing rabbit anti-GFAP (1:2000; Abcam Cat# ab7260, RRID:AB_305808) and mouse anti-NEUN (1:2000; Millipore Cat# MAB377, RRID: AB_2298772). After incubation, the cultures were washed three times with PBS containing 0.1% Triton X-100 for 5 min each. For the next 1.5 h, cultures were stained in blocking solution, containing the secondary antibodies Goat-Antimouse-Alexa488 (1:1000; Thermo Fisher Scientific Cat# A-11029, RRID: AB_2534088), Goat-Antirabbit-Alexa568 (1:1000; Thermo Fisher Scientific Cat# A-11036, RRID: AB_10563566), and DAPI (Biorad, PureBlue DAPI). Then, the cultures were washed with PBS containing 0.1% Triton X-100 and were visualized under a Nikon A1 confocal microscope.

### Statistical analysis

The levels of significance in this study are based on *p*-values calculated using GraphPad Prism 9 (Graph Pad Software). The results are presented as the mean ± standard error of the mean (SEM). The data normality was tested in GraphPad Prism 9 with the Kolmogorov–Smirnov test. Statistical comparisons are specified in Figure captions. A *p* value of <0.05 was considered statistically significant.

## Results

### Evaluation of physiological processes occurring in primary hippocampal cultures during the acute phase of ECM digestion with Hyase

**Figure 1:**
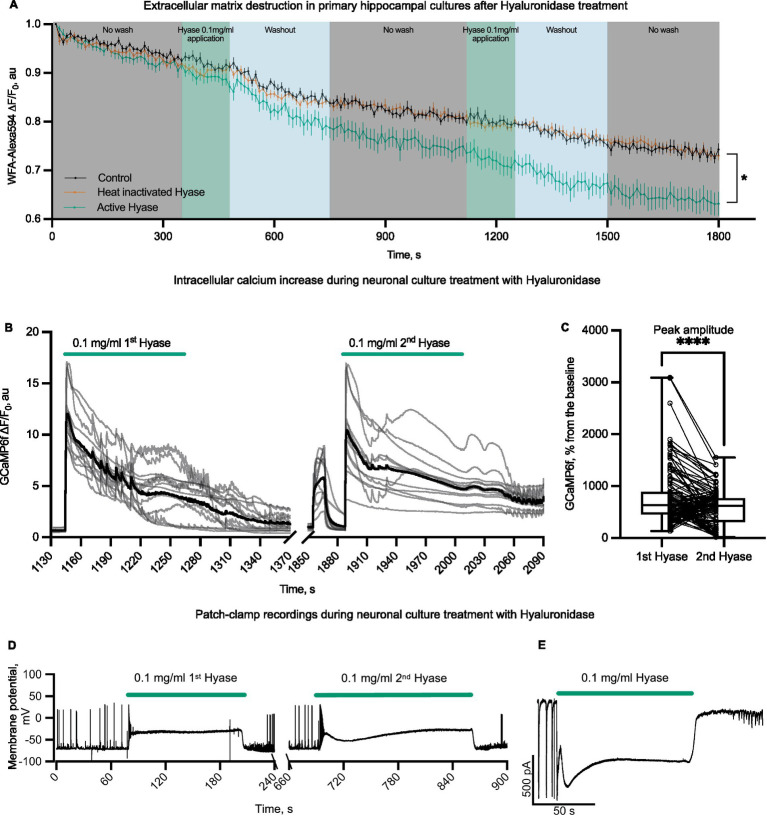
Physiological effects of rapid ECM partial solubilization with hyaluronidase in primary hippocampal cultures. **(A)** WFA-Alexa594 live-stained cells and Hyase applications. The first group (black color on graph, n = 9 from 3 different cultures)—a control group with pure solution application. The second group (vermillion color on graph *n* = 16 from three different cultures)—heat-inactivated Hyase. The third group (green color on graph *n* = 10 from three different cultures)—active hyaluronidase. Graph colors: gray—control acquisition without washing, green—acquisition without washing with the presence of Hyase, blue—washing out with 25 mL of solution. The first hyaluronidase washout contributes to the reduction of the signal from WFA. Two-way ANOVA [*F* (358, 5,760) = 2.889 *p* < 0.0001] with Dunnett’s post-hoc analysis showed significant differences between control and Hyase for points starting from 550 s (550 s, *p* = 0.0385, 1800 s, *p* < 0.0001). The detailed methodology is described in supplementary information. **(B)** Representative traces of neuronal soma GCaMP6f fluorescence with an increased fluorescence during the first and second applications of Hyase for 2 min (green line shows the application of 0.1 mg/mL Hyase). Black line is averaged trace of all cells from one culture. **(C)** GCaMP6f normalized peak amplitudes of the first and second applications of Hyase. Paired comparison of the first and second applications of Hyase by paired t-test, four cultures, 128 neurons. Peak amplitude: Hyase1 = 787.2 ± 47.6% vs. Hyase2 = 573.8 ± 30.8%, *p* < 0.0001. **(D)** Current clamp recordings of membrane potential during the first and second 2-min Hyase applications. Average depolarization during the first application of Hyase was 48.0 ± 4.4 mV, *n* = 13 neurons. **(E)** Voltage clamp recording of membrane current during Hyase application. Hyase concentration specified on graph.

First, we evaluated the presence of the ECM in primary hippocampal cultures. After confirming the presence of the ECM by the *Wisteria floribunda* agglutinin (WFA) fluorescence signal, we tested the ability of Hyase to destroy it (Figure 1A; Supplementary Figure 1A). WFA does not directly bind to hyaluronan but to carbohydrate epitopes on the hyaluronic acid-bounded lectican aggrecan. Therefore, the reduction of WFA is only an indirect indicator of the loss of hyaluronic acid (Nadanaka et al., 2020). We made a control group with pure solution application for the mechanical control, washing control, and control for Alexa594 bleaching. Heat-inactivated Hyase was made to exclude possible side effects on the fluorescent signal. Both groups had a similar drop in fluorescent signal. The third group was active hyaluronidase. We performed two Hyase applications so that our results can be compared to calcium traces. It can be observed that the first Hyase washout contributes to the reduction of the signal from WFA (Nadanaka et al., 2020; Hartig et al., 2022). Next, we examined the physiological consequences of the acute phase of this ECM attenuation. We measured the effects of short Hyase application on cytosolic neuronal calcium levels using the fluorescent calcium sensors, GCaMP6f or OGB1. The administration of Hyase for 2 min resulted in a sharp rise of the fluorescence signal from both these sensors (Figure 1B; Supplementary Figures 1B,C). It is known that Ca^2+^ indicators and BAPTA suppress Na,K-ATPase activity independently from Ca^2+^ chelation and decrease Na,K-ATPase-mediated ion uptake and its ATP hydrolysis (Smith et al., 2018). The genetically encoded calcium sensor GCaMP allows these side effects to be avoided. In our experiments, the genetically encoded calcium sensor GCaMP6f showed results similar to OGB1 (Supplementary Figures 1D,E). The increase in intracellular calcium persisted throughout the presence of Hyase in the extracellular solution. The second Hyase applications after 10 min of washout (the time sufficient for the fluorescence signal returned to the baseline) caused a significantly smaller fluorescence response when compared to the first application, which could be associated with the partial enzymatic cleavage of the ECM after the first Hyase application (Figure 1C). Calcium dynamics in astrocytes was not dependent on Hyase applications (Supplementary Figures 4C,D). Next, we patched neurons expressing GCaMP6f and performed simultaneous recording of calcium dynamics and electrophysiological properties of neurons in the current clamp mode. Hyase was applied locally via micropipette directly to the recorded neurons. The increased fluorescent signal from GCaMP6f was accompanied by membrane depolarization (Supplementary Figure 5A). In most experiments membrane depolarization was rapid, and the cells remained depolarized throughout the drug application period. However, the washout of Hyase led to a rapid restoration of the normal membrane potential of neurons. To exclude possible mechanical and protein–protein interactions, we performed control experiments with bovine serum albumin (BSA), as well as heat-inactivated Hyase. Hyaluronidase inactivated by heating is known to be capable of spontaneous reactivation (Emmart and Longley, 1954). While mechanical control as well as BSA was not able to induce calcium entry, heat-inactivated hyaluronidase application led to short-term intracellular calcium increases. However, analysis showed statistically significant differences between inactivated and active hyaluronidase (Supplementary Figure 2).

**Figure 2:**
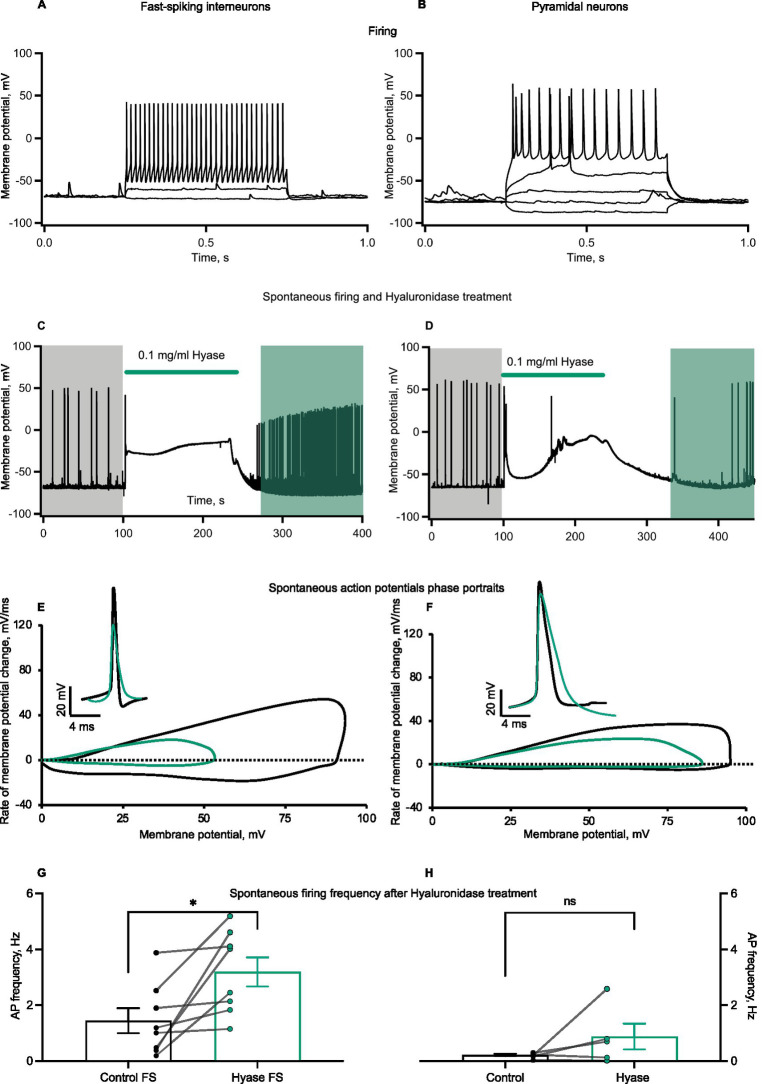
Spontaneous firing and action potential phase portrait of cultured interneurons changes after Hyase treatment. **(A,B)** Representative recordings of firing patterns of cultured fast-spiking interneurons and pyramidal neurons. Action potentials evoked by the depolarization of neuronal membrane with current injections. **(C,D)** Representative traces of spontaneous firing before and after Hyase treatment of fast-spiking and pyramidal neurons. Green line shows the application of Hyase in the recording chamber (around 2 min). Membrane depolarization ascent during Hyase application (FSI 52.7 ± 3.9 mV vs. Pyr 40.5 ± 9.3 mV, *p* = 0.1893, ns) **(E,F)** Phase portraits of spontaneous action potentials before and after Hyase treatment of fast-spiking and pyramidal neurons. Black and green action potentials and phase portraits represent averages before and after Hyase treatment, respectively. **(G,H)** Changes of spontaneous firing frequency after Hyase treatment of fast-spiking and pyramidal neurons. Calculations were performed for the 10 min before and 10 min after Hyase applications (FSI 1.44 ± 0.44 vs. 3.19 ± 0.52 Hz, *p* < 0.0198, *n* = 8; Pyr 0.22 ± 0.043 vs. 0.88 ± 0.46 Hz, *p* = 0.2187, *n* = 5). Hyase concentrations are specified on the graph.

The heterogeneity of neuronal types in primary cultures raised the question of whether different types of neurons respond differently to Hyase exposure. To address this question, we compared responses to Hyase applications from fast-spiking interneurons (FSIs) and pyramidal neurons (Pyrs). Neurons were identified by the action potential (AP) firing patterns triggered by depolarization steps (Figures 2A,B). Both types of neurons showed membrane depolarization during Hyase application (Figures 2C,D). We noticed that FSIs increased their spontaneous AP firing frequency after Hyase washout (Figure 2G). However, the same effect was not observed for Pyrs (Figure 2H). According to the phase portrait, both types of neurons had changes after hyperpolarization, but acute changes in the ECM additionally affected the depolarization phase in FSIs (Figures 2E,F).

### Definition of NMDARs as the target responsible for calcium influx and depolarization during acute matrix destruction with Hyase

Next, we performed combined OGB1 measurements of Hyase-induced calcium influx with pharmacological blockades of possible calcium sources to elucidate how ECM disruption leads to increased cytosolic calcium levels. Surprisingly, we did not see any significant effects on Hyase-induced calcium elevation upon pharmacological suppression of the following channels and signaling cascades: diltiazem (L-VDCCs), BBG (P2X purinoceptor 7), CNQX (AMPARs), dantrolene (ryanodine receptors), 3-Br-7Ni (neuronal nitric oxide synthase), L-NNA (inducible, endothelial nitric oxide synthases), picrotoxin (gamma-aminobutyric acid A receptors), and cyclosporin A (calcineurin/serine/threonine-protein phosphatase 2B) (Supplementary Figure 3).

**Figure 3:**
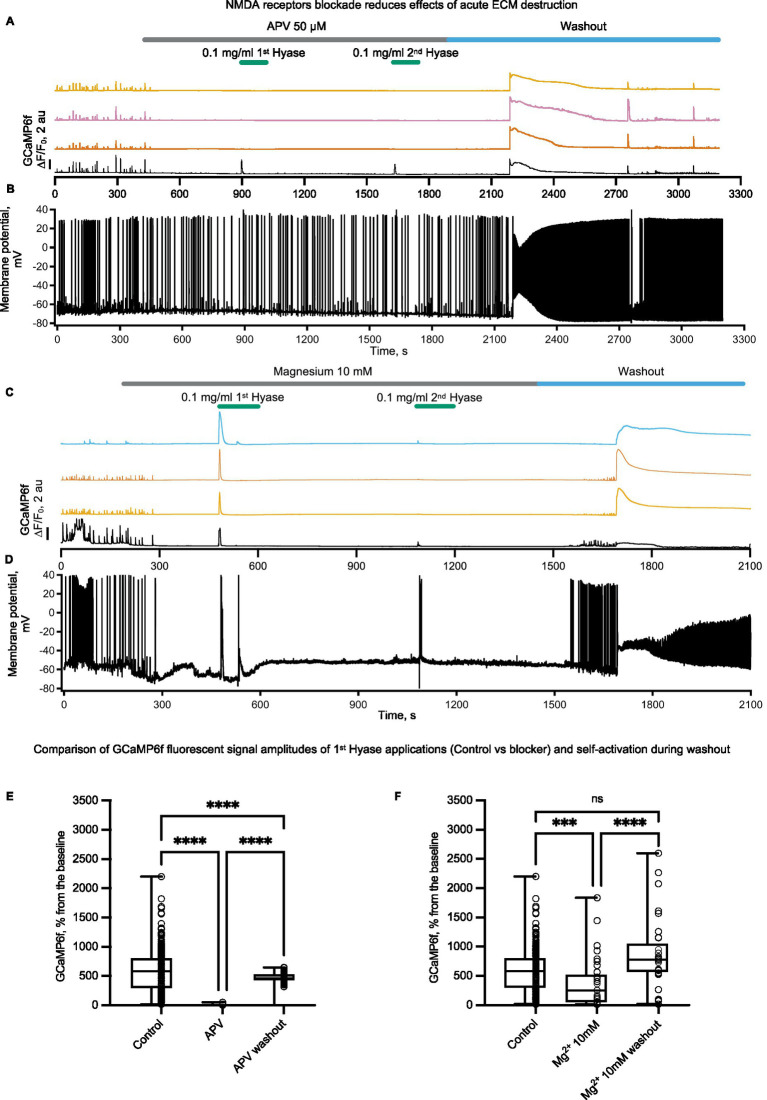
NMDARs blockade prior to rapid ECM digestion by Hyase leads to the disappearance of the prolonged calcium influx and decreases the prolonged membrane potential ascent. Different colors show the signal of GCamp6f from different spontaneously active cells. The patched cell is marked in black color. **(A)** Simultaneous recording of calcium signal and **(B)** membrane potential signal before APV treatment, during two Hyase applications (2 min), and after washout of APV, *n* = 3 cultures. **(C)** Simultaneous recording of calcium signal and **(D)** membrane potential signal before increase of magnesium 10 mM in recording chamber solution, during two Hyase applications (2 min), and after washout with normal magnesium concentration (1.5 mM), *n* = 3 cultures. **(E)** Two-way ANOVA with Tukey post-hoc. Groups consisted of four control cultures (208 cells) vs. 3 APV cultures (64 cells). (Control vs. APV: 597.9 ± 26.7 vs. 5.1 ± 0.8, *p* < 0.0001; APV vs. Washout: 5.1 ± 0.8 vs. 455.9 ± 21.86, *p* < 0.0001). **(F)** Two-way ANOVA with Tukey post-hoc. Groups consisted of four control cultures (208 cells) vs. three Mg^2+^ 10 mM cultures (33 cells). (Control vs. Mg^2+^ 10 mM: 597.9 ± 26.7 vs. 383.7 ± 75.6, *p* < 0.0001; Mg^2+^ 10 mM vs. washout: 383.7 ± 75.6 vs. 868.9 ± 104.1, *p* < 0.0001). Hyase concentrations are specified on the graph.

Hyase-induced calcium entry coincides with strong membrane depolarization. In addition to voltage-gated calcium channels, depolarization in neurons can promote calcium influx through NMDA receptor (NMDAR) channels by relieving them from magnesium block. NMDARs are expressed in all neurons and can be found in extrasynaptic membranes as well as in both postsynaptic and presynaptic sites. Depending on the location, NMDARs are involved in a variety of calcium-dependent processes including synaptic plasticity, regulation of synaptic release, and excitotoxity. Therefore, we decided to explore whether NMDARs can operate as a source for Hyase-triggered calcium entry. The blockade of NMDARs with D-APV showed a significant reduction in both Hyase-induced calcium transients, measured by GCaMP6f, and membrane depolarization (Figures 3A,B,E). Increasing extracellular Mg^2+^ from 1.5 to 10 mM before acute destruction of the ECM led to a shortening of the calcium response to the first application of Hyase and a strong decrease of calcium peak to the second drug administration (Figures 3C,D,F). Interestingly, in both sets of experiments, washout of APV or Mg^2+^ resulted in massive calcium influx with concomitant depolarization even in the absence of Hyase, suggesting that the delayed effects of extracellular matrix breakdown may also be mediated by NMDARs (Figures 3A,C). Previously, an enhancement of neuronal firing and bursting rates specifically through excitatory neurotransmission was reported in Hyase-mediated degradation of the ECM measured with multielectrode arrays (Bikbaev et al., 2015). Cultured neurons from quadruple knockout mice deficient in tenascin-C, tenascin-R, brevican, and neurocan, tested with multielectrode arrays, also showed increased network activity (Gottschling et al., 2019). One reason for this is the impaired endocytosis of NMDARs from the surface rapidly after ECM destruction (Schweitzer et al., 2017).

### Acute phase of ECM destruction increases CA3-CA1 LTP

**Figure 4:**
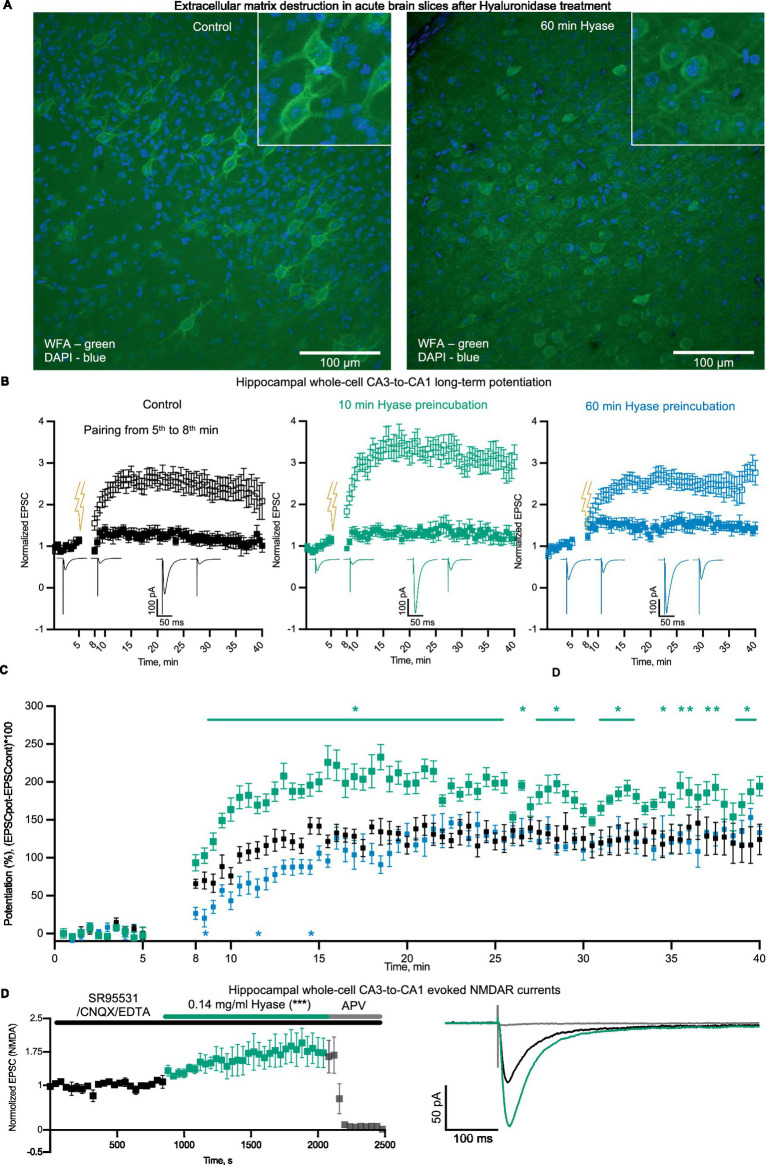
Hyase treatment leads to ECM attenuation and facilitation of synaptic plasticity. **(A)** Confocal images of ECM in acute brain slices labeled with WFA. Left—intact ECM; right—ECM after 60 min of Hyase presence. Scale bar: 100 um. **(B)** Hippocampal whole-cell LTP, paired (open squares) and control inputs (filled squares). Left (black)—Sham hippocampal slices. Middle (green)—10 min preincubation in Hyase. Right (blue)—60 min preincubation in Hyase. **(C)** Normalized potentiation of paired inputs. Groups specified by color. The time window of significantly different time points (two-way ANOVA with Dunnett’s post-hoc analysis) for 10 min incubated slices were from 9th to 40th min after LTP induction. Significantly different timepoints are marked with asterisks. Three significantly different timepoints for 60 min incubated slices marked with asterisks. The n-cells were: control *n* = 7, 10 min Hyase-treated *n* = 7, 60 min Hyase-treated *n* = 5. **(D)** Hippocampal whole-cell EPSCs (*n* = 5), recorded in presence of SR95531, CNQX, and EDTA. Black color shows control EPSCs and green color increasing of EPSCs after Hyase treatment. APV was added to confirm the NMDAR nature of recorded EPSCs. Normalized EPSCs: Control 0.9985 ± 0.0168 vs. Hyase 1.602 ± 0.0359. Unpaired t-test, *p* < 0.0002. Hyase concentration 0.14 mg/mL (for details, see the Materials and Methods section).

The strong stimulation of NMDAR-mediated calcium signaling by ECM disruption suggests that acute ECM breakdown can influence hippocampal synaptic plasticity. First, we tested if Hyase can destroy the ECM in brain slices. WFA labeling of sham slices and slices incubated for 60 min with Hyase showed a reduction in PNN-associated fluorescence in the Hyase-treated slices (Figure 4A). We estimated the acute phase as the beginning of ECM dissociation, where abundant calcium influx was present. For NMDAR-dependent LTP induction, we used a pairing protocol (Rozov et al., 2012). In untreated slices, pairing induced a gradually increasing potentiation of EPSCs that reached a steady-state level of LTP within 20–25 min of induction (Figure 4B). Another set of slices was preincubated in Hyase for 1 h. The induction of LTP, carried out within 20 min after the end of incubation, led to the similar time course of LTP development as in the control, with a small reduction at the initiation phase (Figure 4C). However, a short application of Hyase (10 min) 20 min prior to LTP induction resulted in a significant acceleration of the initial phase of LTP, and this increase was maintained throughout the recording (Figure 4C). The involvement of NMDARs in the Hyase-enhanced LTP was then evaluated. We performed a synaptic transmission assay on the apical pathway, with evoked EPSCs in the presence of an AMPAR blocker. After achieving a stable EPSC baseline for at least 10 min, the addition of Hyase to the ACSF led to a gradual increase in EPSC amplitude. The NMDAR nature of this enhanced EPSC was confirmed by the application of the NMDAR blocker APV, which fully blocked the EPSCs (Figure 4D).

## Discussion

It is considered that the long-term reconstruction of the ECM as a result of neural network events leads to long-term plasticity. Recently, another point of view on ECM remodeling was proposed concerning the existence of a recycling pool of ECM molecules that constantly move between intracellular and extracellular spaces, without the need for novel protein secretion (Dankovich et al., 2021). However, the question of why such a pool of rapid changes in the extracellular space around synapses is necessary remains unanswered. Here, for the first time, we described the acute physiological effects of ECM changes by enzymatic cleavage. We used the classical model of primary hippocampal cultures and present a “novel” target that depends on the rapid ECM changes. We localized the specificity of acute effects during ECM cleavage by hyaluronidase in a population of neurons but not astrocytes. We performed an inhibitory analysis of targets involved in calcium signaling in neurons and confirmed NMDARs as possible contributors to this acute phase. The massive calcium response with simultaneous depolarization, and the delayed effect of ECM destruction observed in our experiments with blocked NMDARs, during washout from NMDAR blocking agents may indicate the existence of a direct connection between NMDARs and the ECM, which was not described earlier. In our experiments with calcium sensors, some cells clearly showed that after the start of Hyase washout, there was another (secondary) increase of Ca^2+^ level (Supplementary Figures 1B,C). It is possible that this increase during washout is of the same nature as the first one and is related to the action of matricryptins. Some authors refer to a downstream cascade from hyaluronic acid receptor CD44 to calcium elevation from intracellular stores via IP3Rs (Singleton and Bourguignon, 2002; Singleton and Bourguignon, 2004). However, we did not observe a similar behavior in cells in the presence of the NMDAR blocker, APV. APV completely blocked the appearance of both types of responses. We assume that, in order to understand the involvement of IP3Rs, it is necessary to focus on other parameters of neuronal activity, such as frequency and amplitude of spontaneous calcium activity after hyaluronidase applications. Another theory of secondary Ca^2+^ increase might be mechanical sensitivity. The fluorescent rise during washout from APV or high magnesium was induced spontaneously, without Hyase application. Piezo-1 is a known mechanosensitive channel present in hippocampal neurons (Falleroni et al., 2022). The influx of calcium via NMDARs during Hyase application can lead to stretching of the neuronal membrane, and ECM shedding may amplify this effect. Thus, we most likely observed both effects simultaneously: the early physiological correlates of hyaluronidase-mediated ECM disintegration alongside a gain of function from the resulting matricryptines.

Previously, the loss of perineuronal nets of FSIs was shown in the peritumoral region. This study indicated the effects of matrix-destroying tumor agents on the biophysical parameters of the membrane, especially the membrane capacitance, affecting the ability of FSIs to generate APs at a high frequency (Tewari et al., 2018). Another study showed a decrease in the number of GABAergic synapses and an increase of synaptic strength after ECM removal *in vitro* (Dzyubenko et al., 2021). While these studies focus on the delayed (hours and days) effects of matrix removal, our study demonstrates the modulation of spontaneous physiological activity at the moment of hyaluronidase enzyme application to the cultured neurons. The phase portraits, obtained from cultured FSIs and Pyrs, highlighted changes in the depolarizing and hyperpolarizing phases. Voltage-gated sodium channels (Na_V_s) are known for their ability to trigger repetitive firing behavior (Tian et al., 1995). Long-lasting Hyase-mediated depolarization can trigger two distinct types of inactivation, termed fast and slow (Goldin, 2003). These types of inactivation prevent neurons from continuous firing during a long depolarization. Increased intracellular calcium concentrations, in addition to prolonged depolarization, can lead to the destabilization of inactivation properties with a significant impact on Na_V_ availability, and therefore on firing frequency and the action potential phase portrait (Van Petegem et al., 2012). While FSIs and Pyrs poorly overlap in the composition of voltage-gated potassium channels (K_V_s), there are general mechanisms of K_V_ regulation (Martina et al., 1998). Many K_V_s undergo slow inactivation upon prolonged depolarization (Pless et al., 2013). Transient K^+^ current can be reduced by the activity of protein kinases, especially protein kinase A and protein kinase C (Johnston et al., 2000; Rudy and McBain, 2001). The activities of these protein kinases are calcium-dependent. Thus, the elevation of intracellular calcium levels due to ECM destruction increases PKA and PKC activities with subsequent disturbance of action potential after hyperpolarization (Dunn et al., 2009; Lipp and Reither, 2011).

Several studies in the hippocampal CA1 region show that ECM degradation decreases LTP (Kochlamazashvili et al., 2010; Bukalo et al., 2001; Saghatelyan et al., 2001; Zhou et al., 2001; Brakebusch et al., 2002; Dityatev et al., 2004; Shi et al., 2019). This has been linked to molecular targets such as tenascin-R, L-VDCC, GABAARs, GABABRs, AMPARs, Brevican, K_V_s, and NMDARs with neuronal cell adhesion molecule (NCAM). On the contrary, there is evidence of increased network activity due to reduced inhibitory activity (Gottschling et al., 2019; Romberg et al., 2013; Lensjo et al., 2017; Wingert and Sorg, 2021). It is considered that PNNs limit the feedback inhibition of parvalbumin-positive (PV+) interneurons to principal neurons, whereas PNN removal results in greater inhibition of these neurons leading to suppression of LTP maintenance. Quadruple knockout mice showed significantly lower levels of GluN2A and GluN2B expression and NMDAR independence in high-frequency stimulation-induced LTP, while this form of LTP was NMDAR-dependent in wild-type mice (Jansen et al., 2017). However, all the above-mentioned studies describe the chronic consequences of global ECM removal. In this article, we explore the effects of short-lasting and, therefore, more physiological modulation of the ECM on neuronal intrinsic properties and synaptic plasticity. We conclude that the acute phase of hyaluronidase application affects both interneurons and pyramidal neurons. We hypothesize that there is activation of NMDA receptors during this phase. At the same time, the enhancement of NMDA-dependent calcium entry during the acute phase of matrix digestion promotes the early phase of long-term potentiation. Increased intracellular calcium concentration resulting in the activation of calcium-dependent LTP-enhancing intracellular signaling proteins or pathways (e.g., CaMK2) may underlie this process. However, ECM destruction together with matrikryptines also diminishes many other plasticity-affecting processes. Thus, the specific role of NMDARs should be further expanded.

## Data Availability

The raw data supporting the conclusions of this article will be made available by the authors, without undue reservation.
