# Supplementary material for: Hyaluronidase-induced matrix remodeling contributes to long-term synaptic changes
Source: Front Neural Circuits. 2025 Jan 17;18:1441280. doi: 10.3389/fncir.2024.1441280 (PMC11782146; doi:10.3389/fncir.2024.1441280)
Supplement: Supplementary file 1 [file Data_Sheet_1.docx]

**Supplementary materials**

Hyaluronidase induced matrix remodeling contributes to long-term synaptic changes

Rostislav Sokolov^a,b^*, Viktoriya Krut’^b,c^, Vsevolod Belousov^b,c,d,e^, Andrey Rozov ^a,c^, Irina V. Mukhina^a,f^

^a^ Institute of Biology and Biomedicine, Lobachevsky State University of Nizhny Novgorod, Nizhny Novgorod, Russia

^b^ Pirogov Russian National Research Medical University, Moscow, Russia

^c^ Federal Center of Brain Research and Neurotechnologies, Federal Medical Biological Agency, Moscow, Russia

^d^ Shemyakin-Ovchinnikov Institute of Bioorganic Chemistry, Russian Academy of Sciences, Moscow, Russia;

^e^ Life Improvement by Future Technologies (LIFT) Center, Skolkovo, Moscow, Russia

^f^ Institute of Fundamental Medicine, Privolzhsky Research Medical University, Nizhny Novgorod, Russia

Rostislav Sokolov

E-mail: sokolov@neuro.nnov.ru

+79601741771

’**Present address:** Pirogov Russian National Research Medical University, Moscow, Russia

Supplementary Figure 1.

(A) Confirmation of the presence of ECM by the *Wisteria floribunda* agglutinin (WFA) fluorescence signal. Representative captures before and after control pure solution (left), heat inactivated Hyase (middle), active Hyase (right).

(B) Representative traces of neuronal somas OGB1 fluorescence with an increased fluorescence during 2 minutes Hyase first and second applications (green line shows the application of Hyase). Black line is averaged trace of all cells from one culture.

(C) Representative traces of neuronal soma GCaMP6f fluorescence with an increased fluorescence during 2 minutes Hyase first and second applications (green line shows the application of 0.1 mg/ml Hyase). Black line is averaged trace of all cells from one culture.

(D) GCaMP6f vs OGB1 F/F0 compairson represented in % from the baseline (Mann-Whitney, GCaMP6f vs OGB1, 597.9± 26.7 vs 125.7± 3.8 p<0.0001).

(E) GCaMP6f vs OGB1 amplitudes of fluorescent signals during hyaluronidase application, normalized on the maximum amplitude of spontaneous activity, recorded during 10 minutes prior hyaluronidase application (Mann-Whitney, GCaMP6f vs OGB1, 134.5±5.3 vs 195.3±17.2 p<0.0001).

Supplementary Figure 2.

(A) Mechanical and protein-protein interactions control with solution and bovine serum albumin (BSA, 0.1 mg/ml). Representative traces of neuronal soma GCaMP6f fluorescence with an increased fluorescence (left). Amplitude comparison (right). Two-way ANOVA with Tukey post-hoc, F (2, 80) = 97.38 p<0.0001; Solution vs. BSA ns, Solution vs. Hyase p<0.0001, BSA vs. Hyase p<0.0001.

(B) Representative traces of neuronal soma GCaMP6f fluorescence after hyaluronidase inactivated by heating and two repetitive applications of active hyaluronidase. Inactivated Hyase can cause small calcium elevation in neurons.

(C) The amplitude of the calcium signal in response to inactivated hyaluronidase is significantly lower than that to active hyaluronidase. The amplitude of calcium signal in response to repeated application of active hyaluronidase did not differ from inactivated hyaluronidase. Previously, we reported that the amplitude of the second response differed and was lower than the first. We explained it as a partial destruction of ECM after the first application. Two-way ANOVA with Bonferroni post-hoc, F (1.556, 118.2) = 54.58 p<0.0001; Heat inactivated Hyase vs. Hyase 1st 0.1mg/ml p<0.0001, Hyase 1st 0.1mg/ml vs. Hyase 2nd 0.1mg/ml p<0.0001, Heat inactivated Hyase vs. Hyase 2nd 0.1mg/ml n.s.).

(D) The pattern of calcium signal shows that in the case of inactivated hyaluronidase there is no prolonged rise of calcium. Therefore, we compared the integral (area under the fluorescent trace curve) of GCaMP6f signal of heat inactivated hyaluronidase with that of active hyaluronidase. The integral was different in all three cases. Two-way ANOVA for repeated measures with Bonferroni post-hoc (F (1.464, 118.6) = 105.5 p<0.0001; Heat inactivated Hyase vs. Hyase 1st 0.1mg/ml p<0.0001, Hyase 1st 0.1mg/ml vs. Hyase 2nd 0.1mg/ml p<0.0001, Heat inactivated Hyase vs. Hyase 2nd 0.1mg/ml p<0.0001).

Supplementary Figure 3.

(A) Ratio of the OGB1 peak amplitude. Second amplitude divided on the first one.

(B) Ratio of the OGB1 averaged amplitude. Second amplitude divided on the first one. Paired comparison of Hyase first and second applications with different blockers (specified on graphs). (C) OGB1 traces with different blockers during first Hyase application. Concentration specified on graph.

Supplementary Figure 4.

(A) Confocal image of E18 hippocampal mixed culture, containing astrocytes and neurons.

(B) Confocal image of P0 hippocampal astrocyte monoculture. Scale bar 100 μm.

(C) Individual traces of GCaMP6s fluorescence signal from astrocytes, co-cultured with neurons. Green line shows the application of Hyase. Hyase concentration specified on graph.

(D) Individual traces of GCaMP6s fluorescence signal from astrocytes, cultured without neurons. Green line shows the application of Hyase. Hyase concentration specified on graph.

**
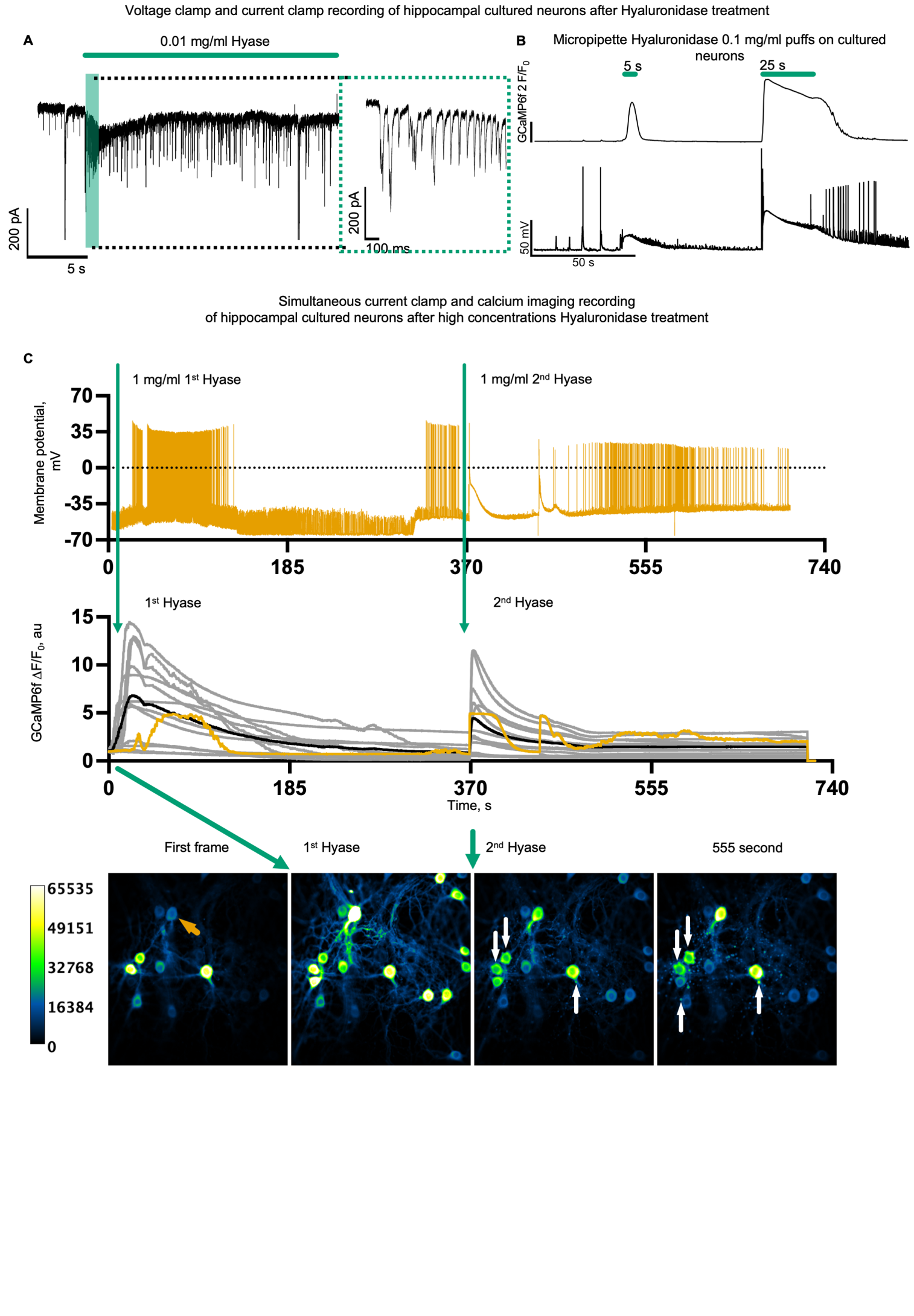
**

Supplementary Figure 5. Hyase concentrations and its effects on neurons.

(A) 10 times decreased Hyase concentration (related to normal one, used in this work) leads to small rapid fast-frequency currents.

(B) Simultaneous recording of GCaMP6f fluorescence and membrane potential in current clamp mode during short term Hyase applications with puff pipette.

(C) 10 times higher Hyase concentration (related to normal one used in this work) leads to huge calcium influx into the neurons and triggers the calcium dependent blebbing of neuronal somas and appendixes (bottom images, white arrows shows blebs of membrane). Orange color on graphs and arrow on images specified patched cell with 0.2 mM EGTA in intracellular solution. EGTA prevented calcium related blebbing. Hyase concentration specified on graph.

**Detailed materials and methods**

*Animal statement*

All experiments followed the European Convention 1986 86/609/EEC, the protocol for cell culture preparations was approved by the ethical committee of the Shemyakin−Ovchinnikov Institute of Bioorganic Chemistry (protocol number 357) and for acute brain slices preparations by the Ethical Committee of Pirogov Russian National Research Medical University. Mice husbandry include breeding pairs in the cage *ad libitum*.

*Cell culture preparation*

Mixed primary hippocampal cell cultures were prepared from E18 embryos or P0 pups. For cell culture preparation and cultivation were used Thermo Scientific mediums and supplements (Gibco, USA). At stage E18, the pregnant C57Bl6 mice were sacrificed by euthanasia, then uterus was dissected out and placed in a sterile Petri dish with cold HBSS solution (Gibco, 14175053). After mild washing from blood the embryos were removed from uterus and also placed in another sterile Petri dish with room temperature HBSS solution (Gibco, 14175053). Embryos were decapitated, hippocampi were dissected from the brain and collected in a tube with pre-heated to 34 °C Versen solution (Gibco, 15040033). Brain tissue was trypsinized with 0.25% solution for 15 min in the incubator. After trypsin removement, the tube with hippocampi on the bottom were rinsed with Plating medium containing DMEM (Gibco 41965039), 10% fetal bovine serum (FBS, Gibco 16140071), 1% gentamicin (Sigma-Aldrich, USA, 1405-41-0). Next cells were washed with 500 ul Plating medium, 4 ul DNAse for 10-15 s. Another wash was made with the Plating medium, then cells were resuspended, and counted in the Goryaev chamber. Round covers coated with polyethylene-imine (PEI, Sigma-Aldrich 408727-100ML) and laminin (Laminin, Sigma-Aldrich, 67407300) were placed into the 24well plate and 5x10^4 cells were placed on each cover. After 25 minutes wells were filled with Full NBM I, containing Neurobasal media (Gibco, 21103049), 0.05% β-mercaptoethanol (Sigma-Aldrich, 60-24-2), 1% Glutamax (Gibco, 35050061), 2% В27 supplement (Gibco, 17504044), 5% FBS, 1% gentamicin. After 7 days remove medium to Full NBM II, containing Neurobasal media, 1% Glutamax, 2% В27 supplement, 0.5% FBS, 1% gentamicin (Sigma-Aldrich, USA). Feed the cultures every 2-3 days. Experimental procedures were started from 21^st^ days *in vitro* (DIV).

Primary astrocyte cultures were prepared from P0 pups, following the same procedures. For astrocyte cultures was used DMEM instead of Neurobasal media.

*Extracellular matrix in vitro recordings*

WFA-Alexa594 was added to the culture media and cultures were incubated at +37C (5% CO2) for 30 min. The final concentration of WFA was 6.5 μg/ml. The solution was removed and cultures were washed with 1 ml of warm HEPES-based solution, after which the dish was filled with 3 ml of HEPES-based solution. Live-cell fluorescent imaging of primary neuronal cell cultures labeled with WFA-Alexa594 was carried out using a Nikon ECLIPSE Ti2-E epifluorescent microscope equipped with a SPECTRA X light engine and a Photometrics BSI camera. The fluorescent signals of AlexaFluor594 were acquired using Plan Apo λ 40x (NA 0.95) objectives. Live cell imaging was carried out using Nikon NIS-Elements software. AlexaFluor594 was excited at a 555 nm, and its fluorescence was acquired within the spectral range 580–610 nm. We performed time-lapse imaging with 10 s interframe intervals and 3s exposition to measure changes in fluorescence in neurons (red positive cells). HEPES-based solution, inactive hyaluronidase, active hyaluronidase was added in a middle of the 10 s interframe interval. Images were captured in 2 randomly selected fields of view for each dish.

*Intracellular calcium recordings and analysis*

For intracellular calcium recordings we used Oregon Green-488 BAPTA-1 AM (OGB1, Invitrogen, USA) or GCaMP6f calcium sensors for neurons. For intracellular calcium recordings of astrocytes, we used GCaMP6s. Used calcium sensor specified on figures and in text.

OGB1 was used for blocker screening. OGB1 was dissolved in dimethylsulfoxide (1 mM stock) and added to the cultures in a wells prior imaging procedure. The finish concentration of OGB1 in well was 1 µM. After 40-min incubation in CO_2_-incubator, covers with cultured cells were submerged in a recording chamber. Recordings were obtained at room temperature (23–25°C). The recording chamber was continuously perfused at 4 ml/min flow with the solution contained (mM): 130-NaCl, 2.5-KCl, 1.5-MgCl2, 1.5-CaCl2 10-glucose, 10-HEPES, pH-7.33 (HEPES-based solution). Neurons were viewed and acquired under water immersion Olympus LUMPLFLN40×W objective with 40X magnification. Evolve512 EMCCD camera (Teledyne Photometrics, UK) was mounted on Scientifica SliceScopePro 2000 (RRID:SCR_018405; Scientifica, UK) microscope. Data acquisition were performed with AxioVision 4.8.2 (Carl Zeiss, Germany) at 3.6 fps. ROIs positioning were performed manually, ROIs were placed on cells somas. For fluorescent signal normalization we have used background ROI function of Physiology acquisition module of AxioVision 4.8.2. Signal of the background ROI was subtracted from signal of cells ROIs. Then all given signal values were divided by the value from the first image of time stack. Thereby the OGB1 ratio signal was received. Calcium signal were analyzed using Clampfit (pClamp, RRID:SCR_011323, Molecular Devices, USA) software. The baseline of Ca2+-sensitive fluorescence for each cell at physiological rest was taken as a 100% reference point. Thus, each effect is represented as n% increase from the baseline. All pharmacological agents were purchased from Sigma-Aldrich or Tocris Bioscience. Oregon Green-488 BAPTA-1 AM was used as the cell-permeable photosensitive fluorescent probe with a rather low dissociation constant for Ca2+ (Kd ~ 170 nM) and high fluorescence intensity, according to the supplier datasheet.

To express GCaMP6f calcium sensor in neurons or GCaMP6s calcium sensor in astrocytes *in vitro* were used created recombinant adeno-associated viral (AAV) vectors serotype DJ and 9, carrying the respective genetic construct. AAVs were added to cultures at 7-10 DIV, in 10000 viral units/cell concentration. Recordings were obtained at room temperature (23–25°C). Neurons were viewed and acquired under water immersion Olympus LUMPLFLN40×W objective with 40X magnification. ORCA Flash4.0 LT plus digital sCMOS camera (RRID:SCR_021971, Hamamatsu Photonics) was mounted on Scientifica SliceScopePro 2000 (RRID:SCR_018405; Scientifica, UK) microscope. Data acquisition and ROI positions were performed with free software uManager (RRID:SCR_000415) at 20 fps rate. Light source used in these experiments was CoolLED pE-300ultra (RRID:SCR_021972) synchronized with patch-clamp recordings via BNC-TTL output from Heka Elektronik EPC 10 USB Patch Clamp Amplifier. Offline measurements of time-dependent changes in intensity of fluorescence were performed with Fiji software (RRID:SCR_002285). It was used to create quantitative datasets for determining the kinetics of GCaMP6f in cultures. Cell bodies with fluorescence were selected as regions of interest (ROIs) and added to the ROI manager of Fiji software. The background ROI was selected on the field of view without the valuable fluorescent signal from cells or appendixes. Mean values of fluorescence for ROI were measured, and digitized fluorescence datasets for each ROI in gray channel were exported using the Multi measure tool. Signal of the background ROI was subtracted from signal of cells ROIs. Then all given signal values were divided by the value from the first image of time stack. Thereby the GCaMP6f ratio signal was received.

*Preparation of acute brain slices*

Males C57Bl6 mice were deeply anesthetized with 3% isoflurane and decapitated, and brains were isolated from skulls. Transverse hippocampal 300 μm slices were prepared from the isolated brains. The slicing chamber contained an oxygenated ice-cold solution composed of (in mM): K-Gluconate, 140; N-(2-hydroxyethyl) piperazine-N′ -ethanesulfonic acid (HEPES), 10; Na- Gluconate, 15; ethylene glycol-bis (2-aminoethyl)-N, N, N′ , N′ -tetra- acetic acid (EGTA), 0.2; and NaCl, 4 (pH 7.2). Slices were incubated for 30 min at 35 ◦C before being stored at room temperature in artificial cerebro-spinal fluid (ACSF) containing (in mM): NaCl, 125; NaHCO3, 25; KCl, 2.5; NaH2PO4, 1.25; MgCl2, 1; CaCl2, 2; and D-glucose, 25; bubbled with 95% O2 and 5% CO2.

*Electrophysiological procedures*

Electrophysiological recordings of cultured neurons were conducted at 21-26 DIV. Neurons were viewed under DIC or epifluorescence illumination at 40X magnification. Patch electrodes were pulled from hard borosilicate capillary glass (Sutter P-97/PC Pipette Puller (RRID:SCR_018636)). Whole-cell patch-clamp recordings were obtained at room temperature (23–25°C) using pipettes (2–3 MΩ), filled with (mM): 130-K-gluconate, 1-MgCl_2_, 3-L-ascorbic acid, 10-HEPES, 2.5-Na_2_ATP, 1-Na_3_GTP, 295±3 mOsm, pH-7.35. The recording chamber was perfused with HEPES-based solution. Heka Elektronik EPC 10 USB Patch Clamp Amplifier (RRID:SCR_018399) was connected to a computer running Patchmaster software (RRID:SCR_000034). Data were collected with a 10 kHz digitization rate in current-clamp mode. Acceptable cells had resting potentials greater than −60 mV. Cells with rundown more than 100 pA were excluded from the analysis.

Electrophysiological recordings from acute brain slices During experiments, slices were continuously perfused with ACSF. Electrodes for the postsynaptic pyramidal cells were filled with a solution consisting of (in mM): Cs-gluconate, 136; CsCl, 4; HEPES, 10; NaCl, 8; MgATP, 4; MgGTP, 0.3; phosphocreatine, 10 (pH 7.3 with CsOH). Whole-cell recordings from neurons were conducted at room temperature (23–25 °C) in voltage-clamp mode using a Heka Elektronik EPC 10 USB Patch Clamp Amplifier (RRID:SCR_018399) connected to a PC running Patchmaster software (RRID:SCR_000034). Data were collected with a 10 kHz digitization rate in voltage-clamp mode filtered at 3 kHz. CA1 pyramidal cells were visually identified using IR-video microscope Scientifica SliceScopePro 2000 (RRID:SCR_018405; Scientifica, UK) under DIC at 40X magnification. GABAergic synaptic transmission was blocked by continuous presence of the GABAA receptor antagonist SR95531 (10 μM). In LTP procedures, two patch pipettes were used as stimulating electrodes in *stratum oriens* and *stratum radiatum*, respectively. EPSCs were evoked from two independent inputs, to the basal and apical dendrites. The input to the apical dendrites was potentiated, whereas the input to the basal dendrites acted as the control pathway. Neurons were maintained at −70 mV before and after LTP induction, and both inputs were stimulated every 6 s. LTP induction was made by pairing depolarization of the postsynaptic patched pyramidal neuron to 0 mV for 3 minutes simultaneously with stimulation of the inputs to the apical dendrites every 1.5 s. The measured amplitudes were normalized to the mean baseline EPSCs before pairing. For NMDARs evoked EPSCs was measured in the apical pathway. We used ACSF without magnesium, supplemented with 50 μM magnesium chelator EDTA. The baseline was measured in the presence of GABAA receptor antagonist SR95531 (10 μM) and AMPARs blocker CNQX. After Hyase treatment and recordings NMDARs EPSCs were blocked with APV.

*Confocal imaging of Wisteria Floribunda agglutinin-stained cultures and brain slices*

We imaged cultures and slices using an inverted Nikon A1 confocal microscope. To confirm the presence of ECM we labeled cultures and slices with *Wisteria Floribunda* agglutinin known to selectively bind to N-acetylgalactosamines beta 1 (WFA, Sigma-Aldrich, L8258-5MG). Due to unlabeled WFA, we conjugated it with FITC (Lumiprobe, 3524-500mg). For WFA conjugation with FITC we used QIAquick Spin Columns (QIAGEN GmbH, Germany).

*Slice preparations for WFA staining. WFA staining.*

For brain tissue dissection, fixation and slicing, prior WFA slice staining, mice were transcardially perfused with 4% PFA. Then brains were dehydrated by sucrose gradient. 50 μm slices were cut on a cryotome HM525 NX (Thermo Scientific, USA) and stored in PBS-azide solution. For staining, slices were incubated in 25 mM glycine solution 20 minutes and then washed three times with PBS. Then Hyase was added to slices for 1 hour. Next slices were washed three times with PBS. Slices were initially permeabilized in PBS with 0.5% Triton X-100 (Sigma-Aldrich, #T8787) for 1 h. Blocking was made in PBS containing 5% goat serum (Sigma- Aldrich, #G9023) and 0.1% Triton X-100 next 1 h. Then, slices were stained with the WFA-FITC (20 ug/ml) diluted in the blocking solution (0.1% triton, 1% goat serum) for 48 hours under +4 °C conditions. Slices were washed three times with PBS (5 min each), mounted on slides and visualized under the confocal microscope.

*Primary cultures immunostaining with antibodies.*

Immunostaining was performed since 24^th^ DIV. Cultures were washed with PBS (35°С). Next cultures were fixed for 15 min with fresh 4% PFA on the room temperature. After fixation cultures were washed 3 times with PBS 5 min each. Permeabilization was performed in PBS with 1% Triton X-100 for 15 min. Blocking was made in PBS containing 5% goat serum (Sigma- Aldrich, #G9023) and 0.1% Triton X-100 next 1 h. Then cultures were washed with PBS and 0.1% Triton X-100. Primary antibody labeling was made next 12 hours at +4 °С in blocking solution, containing rabbit Аnti-GFAP (1:2000; Abcam, Ab7260) and mouse Anti-NEUN (1:2000; Millipore, MAB377). After incubation cultures were washed 3 times with PBS containing 0.1% Triton X-100 5 min each. Next 1.5 h cultures were stained in blocking solution, containing secondary antibodies Goat-Antimouse-Alexa488 (1:1000; Thermo Scientific, A-11029), Goat-Antirabbit-Alexa568 (1:1000; Thermo Scientific, A-11036), and DAPI (Biorad, PureBlue DAPI). Then cultures were washed with PBS containing 0.1% Triton X-100. Cultures were visualized under the confocal microscope Nikon A1.

*Construction of vectors for expression of GCaMP6f,s*

To express the GCaMP6f in neurons *in vitro* and GCaMP6s in astrocytes *in vitro*, we created recombinant adeno-associated viral (AAV) vectors, carrying the respective genetic constructs.

The plasmid contains hSyn promoter - the promoter of the human gene synapsin 1, was gifted from the Penn Vector Core (Addgene plasmid #100848). The plasmid contains GCaMP6f was gifted from Balijit Khakh (Addgene plasmid #52925).

The construct pAAV-hSyn-GCamp6f-SV40 were created on the basis of the pAAV.Syn.NES.jCaMP1a.WPRE.SV40 and pZac2.1gfaABC1D-cyto-GCaMP6f by replacing a promoter and fusing the corresponding cDNAs in the same open read frame. All manipulations were conducted between the NheI and MfeI restriction enzyme sites. Escherichia coli XL1-Blue strain (Evrogen, #CC001) was used for the cloning, maintenance, and propagation of plasmids. For limit the expression of GCaMP6s only by astrocytes, we used a recombinant adeno-associated vector of the 9th serotype carrying the construct pAAV-GFAP-GCamp6s-(wpre-sv40), where GFAP is a promoter of glial fibrillar acid protein. Gene encoding GCaMP6s was amplified from plasmids previously generated in our laboratory. Midipreps were prepared using the QIAGEN Plasmid Midi Kit (Qiagen, 12145) according to the manufacturer’s instructions. The constructs were packaged into serotype 9 AAV viral particles for astrocytes and serotype DJ for neurons at the Viral Core Facility of Shemyakin-Ovchinnikov Institute of Bioorganic Chemistry.

The virus titers were 2.8E+13 viral genomes (VG)/mL for AAV2/DJ-hSyn-GCaMP6f; 1.1E+13 VG/mL for AAV2/9-GFAP-GCaMP6s.

*Extracellular brain matrix destruction by Hyaluronidase treatment of cultures and slices*

For ECM destruction we used Hyaluronidase from bovine testes (Hyase, Sigma-Aldrich, H3506-1G, 400-1000U/mg). These enzymes known to degrades hyaluronan and cleave glycosidic bonds in hyaluronic acid, chondroitin, and chondroitin sulfates (according to Sigma-Aldrich H3506-product description). Working Hyase solution for cultured neurons was prepared from HEPES-based solution. Working Hyase solution for slices was prepared from ACSF. Solutions were aliquoted and frozen, according with Sigma-Aldrich recommendations. Solutions contained 50 mg/ml what was equal from 20000 to 50000 units of activity (U) in milliliter (20-50 U/µl). After aliqute defrosting, 10 µl (0.5mg) of Hyase solution for cultures was dissolved in 5ml of HEPES-based fresh solution to reach the final concentration of 40-100 U/ml (0.04-0.1 U/µl, 0.1mg/ml) and were rapidly added to recording chamber with culture (or added to the puff pipette for further direct puffs). For simplification, this concentration will be indicated further on the figures and in the text as 0.1 mg/ml. Working concentrations for rapid ECM digestion in experiments with acute brain slices was taken as ~0.1 U/µl. Prior aliquot defrosting, submerged incubation chamber for only 2-3 slices and 35 ml volume we filled with ACSF and oxygenated. Slices were moved to the chamber. After aliqute defrosting, 100 µl of Hyase (2000-5000 U) solution for slices was dissolved in 35 ml of submerged incubation chamber to reach the final concentration of ~57-143 U/ml (~0.06-0.14 U/µl, 0.14 mg/ml). The same concentrations of Hyase (0.04-0.1 U/µl for cultures and ~0.06-0.14 U/µl for slices) were used for ECM destruction before WFA staining.

*Statistical Analysis*

Levels of significance in this study are based on p-values calculated by GraphPad Prism 9 (Graph Pad Software). The data normality was tested in GraphPad Prism 9 with the Kolmogorov-Smirnov test.

Analysis of fluorescent calcium sensors was performed only in neurons with neuronal spontaneous calcium activity. Figure 1B, C F/F0 Mean±Std.ErrorOfMean values of GCaMP6f % from a baseline. Comparison of Hyase first application and second application compared by Wilcoxon non-parametric paired test, Two-tailed Exact P value provided in Results section.

Analysis of spontaneous AP firing performed from n-neurons with paired t-test with two-tailed P-values specified in Results section and on graph.

The statistical significance of LTP for slices that received Hyase treatment under different experimental conditions was assessed by comparing normalized EPSC amplitudes in the paired apical and unpaired basal pathways recorded in the period 0–30 min after LTP induction. For cross-comparison effects of treated and untreated neurons, the values of relative potentiation calculated by subtraction of the normalized EPSC amplitudes in the unpaired basal pathway from those in the paired apical pathway were used. The time window of significantly different time points (Two-way ANOVA with Dunnett's post-hoc analysis) are marked with asterisks. The n-cells are given on the graph.
