# Supplementary material for: Hyaluronidase-induced matrix remodeling contributes to long-term synaptic changes
Source: Front Neural Circuits. 2025 Jan 17;18:1441280. doi: 10.3389/fncir.2024.1441280 (PMC11782146; doi:10.3389/fncir.2024.1441280)
Supplement: Supplementary file 2 [file Table_1.docx]

**Supplementary Table 1.** Names of used chemicals with the catalogue number.

| **Name** | **Cat.#** |
| --- | --- |
| Gibco HBSS solution | 14175053 |
| Gibco Versen solution | 15040033 |
| Gibco DMEM media | 41965039 |
| Gibco fetal bovine serum | 16140071 |
| Sigma gentamicin | 1405-41-0 |
| Sigma polyethylene-imine | 408727-100ML |
| Sigma laminin | 67407300 |
| Gibco Neurobasal media | 21103049 |
| Sigma β-mercaptoethanol | 60-24-2 |
| Gibco Glutamax | 35050061 |
| Gibco В27 supplement | 17504044 |
| Invitrogen Oregon Green-488 BAPTA-1 AM | O6807 |
| Applichem Sodium Chloride | 141659 |
| Applichem Potassium Chloride | A2939,1000 |
| Applichem Magnesium Chloride 6-hydrate | 141396 |
| Applichem Calcium Chloride | A3652,1000 |
| Sigma HEPES | H3375-250G |
| Applichem D-glucose | 141341 |
| Sigma Potassium gluconate | P1847-100G |
| Sigma D-gluconic acid sodium salt (Na-Gluconate) | G9005-500G |
| Sigma EGTA | E4378-25G |
| Sigma Sodium bicarbonate (NaHCO3) | S5761-1KG |
| Sigma Sodium phosphate monobasic (NaH2PO4) | S5011-1KG |
| Sigma L-Ascorbic acid | A4544-25G |
| Sigma GTP sodium salt hydrate | 51120-100MG |
| Sigma Cesium chloride | C4036-100G |
| Hellobio Cesium Gluconate | HB4822-10G |
| Sigma Adenosine 5′-triphosphate magnesium salt | A9187-1G |
| Sigma Phosphocreatine disodium salt hydrate | P7936-5G |
| R&D systems SR95531 hydrobromide | 1262/50 |
| Sigma *Wisteria Floribunda* agglutinin | L8258-5MG |
| Lumiprobe FITC | 3524-500mg |
| Sigma paraformaldehyde | 30525-89-4 |
| Sigma Triton X-100 | T8787 |
| Gibco PBS pH 7.4 (1X) | 100100234 |
| Sigma goat serum | G9023 |
| Sigma Hyaluronidase from bovine testes | H3506-1G |
| Tocris 3-Bromo-7-nitroindazole | 0735 |
| Sigma Nω-Nitro-L-arginine | N5501 |
| Tocris Cyclosporine A | 1101 |
| Sigma bovine serum albumin | A7906-100G |
| Sigma diltiazem | 309866 |
| Sigma Brilliant Blue G | B0770 |
| Sigma CNQX disodium salt hydrate | C239-5MG |
| Tocris dantrolene sodium salt | 0507 |
| Tocris picrotoxin | 1128 |
